# Lobaplatin hyperthermic intraperitoneal chemotherapy plus cytoreduction and rechallenge using cetuximab for wild-type RAS peritoneal metastatic colon cancer: a case report and literature review

**DOI:** 10.1186/s12876-022-02109-z

**Published:** 2022-02-14

**Authors:** Peilin Dai, Zaisheng Ye, Zhai Cai, Zeyu Luo, Enming Qiu, Yu Lin, Jian Cai, Hui Wang, Zhou Li, Shuai Han

**Affiliations:** 1 Second Clinical Medical College of Southern Medical University, Guangzhou, 510515 Guangdong China; 2 Department of Gastrointestinal Surgical Oncology, Fujian Cancer Hospital and Fujian Medical University Cancer Hospital, Fuzhou, 350000 Fujian China; 3 General Surgery Center, Department of Gastrointestinal Surgery, Zhujiang Hospital of Southern Medical University, Guangzhou, 510280 Guangdong China; 4 Department of Pathology, Zhujiang Hospital of Southern Medical University, Guangzhou, 510280 Guangdong China; 5 Department of Colorectal Surgery, The Sixth Affiliated Hospital, Sun Yat-Sen University, Guangzhou, 510000 Guangdong China; 6 Guangdong Provincial Key Laboratory of Colorectal and Pelvic Floor Diseases, The Sixth Affiliated Hospital, Sun Yat-Sen University, Guangzhou, 510000 Guangdong China

**Keywords:** Lobaplatin, Hyperthermic intraperitoneal chemotherapy and cytoreductive surgery, Peritoneal metastatic colon cancer, Rechallenge, Case report

## Abstract

**Background:**

Synchronous peritoneal metastasis of colorectal cancer usually predicts a bleak prognosis. Hyperthermic intraperitoneal chemotherapy (HIPEC) and cytoreductive surgery (CRS) have brought a glimmer of hope to the treatment of peritoneal cancer. Few cases treated with lobaplatin have been reported in the literature and the regimen is controversial. In this case, the comprehensive treatment scheme of lobaplatin-based HIPEC plus CRS and rechallenge using cetuximab plus systemic chemotherapy is effective, especially for the patients with left colon cancer (wild-type RAS).

**Case presentation:**

A 49 year-old man with signet ring cell carcinoma of sigmoid colon with extensive abdominal metastasis (wild-type RAS) was hospitalized with prolonged abdominal pain, distention and abdominal mass. After receiving HIPEC with lobaplatin and XELOX regimen combined with cetuximab for eight cycles, the patient had been treated with the FOLFIRI regimen and cetuximab for 24 cycles, which discontinued due to myelosuppression. Because the disease recurred unfortunately 4 months later, the FOLFIRI + cetuximab regimen was initiated again and stopped after two cycles. Intestinal obstruction occurred 1 month later, so open total colectomy, CRS + HIPEC and ileorectal anastomosis were performed. Capecitabine adjuvant chemotherapy was administered, followed by the maintenance therapy with FOLFIRI + cetuximab regimen. After that, the patient has been in relatively stable condition. By August 2021, the overall survival is more than 45 months, which displays significant curative effect.

**Conclusion:**

For peritoneal metastasis from left colon cancer, the management with CRS + lobaplatin HIPEC and rechallenge of systemic chemotherapy plus targeted medicine based on gene detection can dramatically improve prognosis and extend the overall survival.

## Background

As one of the most common malignant tumors, colorectal cancer (CRC) ranks third in terms of incidence and second in mortality [1]. Peritoneal metastasis (PM) occurs in 5–15% of patients with initial diagnosis of synchronous metastatic colorectal cancer (mCRC) [2, 3]. Peritoneal carcinoma (PC) often indicates unsatisfactory prospects and prognosis in metastatic or stage IV colorectal cancer [4, 5]. For colon cancer, it used to be considered that the presence of implanted tumor foci in the peritoneum suggests the advanced stage of the tumor, and palliative treatment was adopted without effective clinical measures. The therapy combined hyperthermic intraperitoneal chemotherapy (HIPEC) with cytoreductive surgery (CRS) is the most effective strategy for the treatment of PC by removing the visible cancer tissues of peritoneum and abdominal and pelvic cavity through CRS, and then removing the residual micro cancer foci through the synergistic effect of HIPEC thermochemotherapy. However, there are disputes about this treatment due to its correlation with high recurrence rate and toxic side effects [6]. Furthermore, though several authoritative guides are practical [7], the individual heterogeneity of tumor location and genotype contribute to the diverse specific regimens in terms of drug regimen, infusion volume, duration and PM concentration [8, 9], which also increased the uncertainty of the curative effect.

As one of the new generation of platinum, lobaplatin’s inhibitory effect on CRC cells is similar to oxaliplatin. However, compared with oxaliplatin, it only shows the specific side effect on platelet inhibition. If the patient only receives intraperitoneal chemotherapy, the adverse reaction will be mild. Therefore, it is theoretically more suitable for intraperitoneal chemotherapy [10–12]. But few cases treated with lobaplatin have been reported in the literature so far. The application of targeted drugs based on gene detection is an important treatment strategy for colon cancer. RAS and BRAF in EGFR signaling pathway are classical molecular markers of mCRC [13], playing an important role in the survival and proliferation of tumor cells. Cetuximab,the representative drug of EGFR monoclonal antibody, combined with chemotherapy is the first-line treatment for RAS-wt metastatic colorectal cancer. Although disease progression is inevitable, rechallenge and maintenance treatment with cetuximab may be beneficial [14]. Here we present the case of a patient who received lobaplatin HIPEC + CRS, ileorectal anastomosis and chemotherapy and cetuximab rechallenge. Fortunately, after a series of treatment, his condition returned to stability. The comprehensive therapy of lobaplatin-based HIPEC + CRS, chemotherapy and cetuximab rechallenge is highly beneficial to the overall survival and improvement of peritoneal metastasis of RAS-wt colon cancer. As far as we know, this is the first case report of sigmoid colon with extensive abdominal metastasis (wild-type RAS) implementing HIPEC with specific dose of lobaplatin plus cytoreductive therapy. The aim is to provide a valuable reference for the treatment of similar patients in clinic based on the significant therapeutic effect of lobaplatin in this case.

## Case presentation

A 49-year-old male, businessman, was admitted to the Department of Gastrointestinal Surgery for prolonged abdominal pain and distention lasting for 5 months. A left lower abdominal mass had been found for 1 week. There was no obvious inducement for his stabbing abdominal pain for 5 months, which was intermittent and progressive, accompanied by intermittent bloody stool. The frequency of defecation increased, and the patient discharged unformed loose stool, with feeling of incomplete defecation and constipation. The symptoms above are accompanied by fatigue, weight loss, loss of appetite, but without fever, nausea and vomiting. Through abdominal palpation, there is a hard mass in the left lower abdomen. The abdomen is slightly elevated, with slight tenderness and no rebound pain. No obvious abnormality was found in other physical examinations. The patient was in good health before, with no smoking and drinking history. There had been similar patient in his family.

**Fig. 1:**
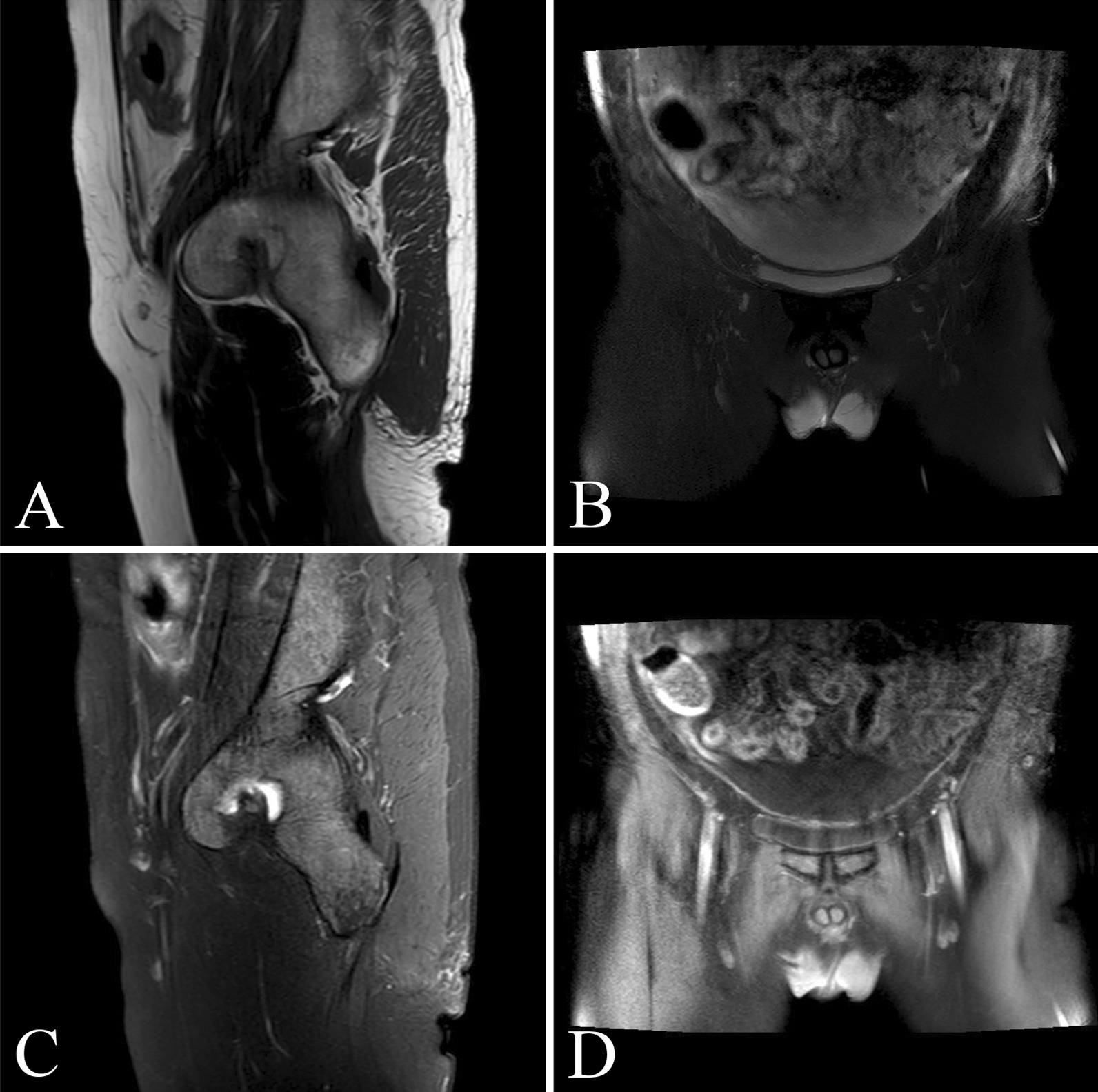
Magnetic resonance images of lower abdomen and pelvis. MRI showing the local intestinal wall of sigmoid colon was thickened (**a**, **c**), the peritoneum was unevenly thickened, and there was liquid signal shadow in pelvic cavity (**b**, **d**)

**Fig. 2:**
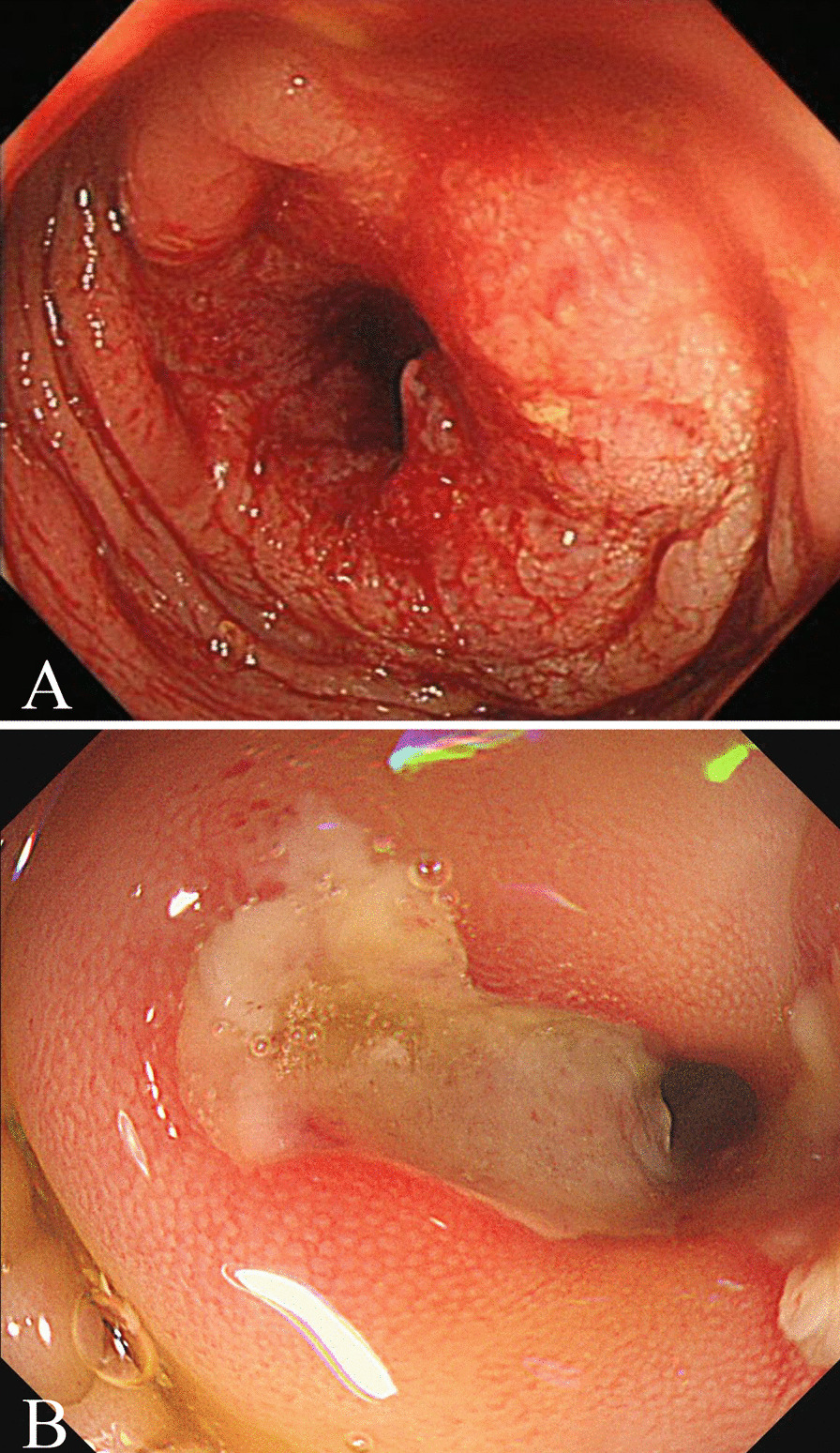
Colonoscopy image comparison. Before treatment: **a** Colonoscopy revealed rigidity and erosion of intestinal wall, and thickened intestinal wall at the middle segment of rectum causing an obvious stenosis of enteric cavity; After 24 cycles of the cituximab and FOLFIRI regimen: **b** Colonoscopy showed that the bleeding and erosion of intestinal wall were significantly improved

**Fig. 3:**
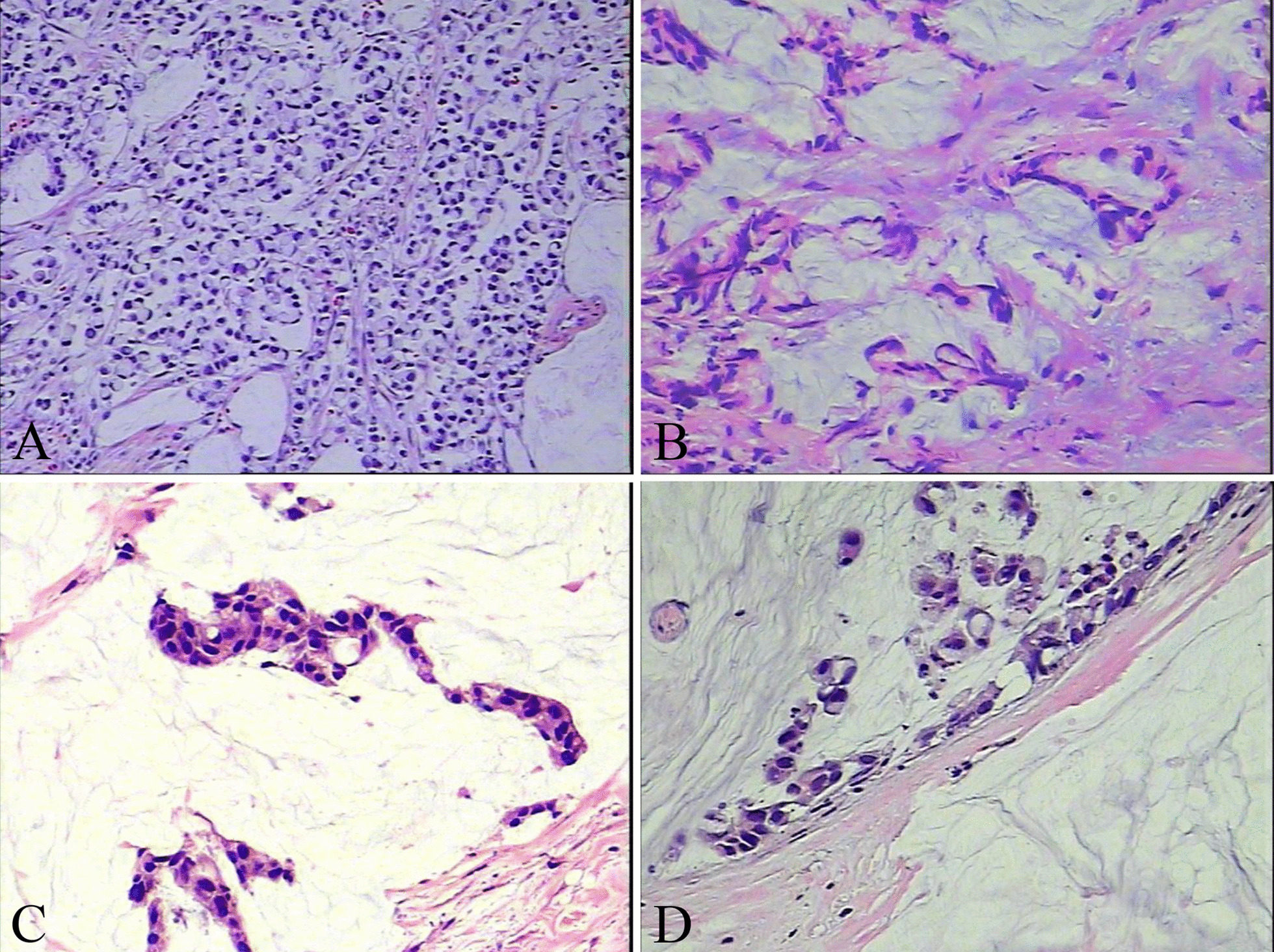
The histopathological findings. The cancer tissue of sigmoid colon was arranged in a patchy irregular adenoid pattern, and most of the cells were signet ring like, with rich cytoplasm, deeply stained nuclei and deviation; the cancerous tissue of greater omental nodule had fibrous hyperplasia, which was adenoid and arranged in a cord shape. The nuclei of cancer cells were deeply stained and mitotic (**a**, **b** Hematoxylin–Eosin, 100×). The cancer tissue of mesenteric nodule floated in a mucinous lake, with sparse cytoplasm and obvious nuclear atypia (**c**, **d** Hematoxylin–Eosin, 200×)

**Fig. 4:**
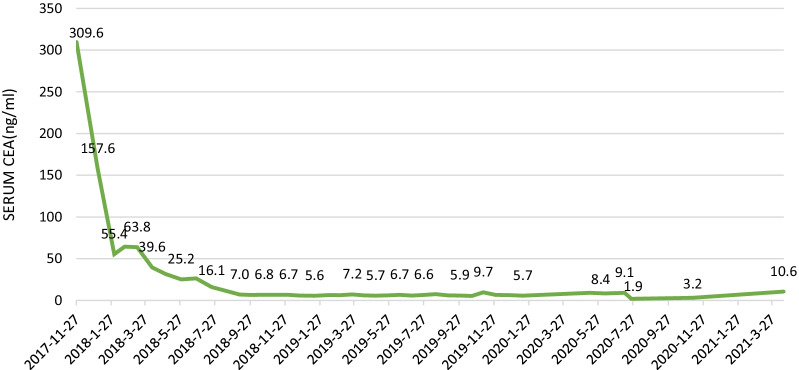
The trend of CEA concentration. The CEA concentration showed a sharp decrease during the cetuximab (600 mg) and the XELOX regimen; the curative effect was even more remarkable (under 10 ng/ml) by using the FOLFIRI and cetuximab regimen (800 mg). The CEA concentration remained relatively stable in the later period

Computed tomography (CT) of the chest, abdomen, and pelvis was performed and confirmed that there was a strip shadow in the anterior segment of the upper lobe of the right lung and a large amount of effusion in abdominal and pelvic cavity, and the local intestinal wall of sigmoid colon was thickened with elevated blood levels of carcinoembryonic antigen (CEA) of 309.6 ng/ml and CA19-9 of 239.0 u/ml. Magnetic resonance imaging (MRI) showed the similar results, and the peritoneum and local sigmoid colon were significantly enhanced on enhanced scanning (Fig. 1). Colonoscopy revealed that the intestinal cavity was stiff and erosive from 2 to 30 cm away from the anal margin, involving the whole intestinal cavity. The sigmoid colon with circumferential uplift was brittle and easy to bleed, and the intestinal cavity was slightly narrow. A subsequent colonoscopic biopsy revealed a signet cell carcinoma of colon (Fig. 2), and the immunohistochemical parameters confirmed: CK(+), CK20(+), CDX-2(+), CEA(+), Ki-67 about 80% (+). The patient was healthy before, but has positive familial history. After discussion, the multidisciplinary team (MDT) considered laparoscopic abdominal exploration. In the operating field, there were a large number of pale yellow bloody ascites in the abdominal cavity, multiple metastatic nodules or planting metastases in the peritoneum, liver, mesentery and pelvic cavity, and the rigid sigmoid colon and mesangium were unable to be dissociated under endoscopy. Resection of sigmoid colon at the first operation was likely to accelerate systemic metastasis of cancer cells after operation. So the patient underwent laparoscopic resection and biopsy of metastatic nodules, and intraperitoneal thermal perfusion chemotherapy after operation. Referring to the pathological results (Fig. 3), the patient was finally diagnosed as signet ring cell carcinoma of sigmoid colon with extensive abdominal metastasis (pT4bNxM1c stage IV). Then, the patients were treated with HIPEC (20 mg lobaplatin and 3 L normal saline at 43 °C for 60 min by specific dual circulation instruments), once a day for 5 consecutive days. According to the results of molecular pathological examination, there was no mutation at the common mutation sites of KRAS gene. Then cetuximab (600 mg) and the XELOX chemotherapy regimen comprising oxaliplatin (130 mg/m^2^) and capecitabine (1000 mg/m^2^), 21 days per cycle, had been applied to this patient for 8 cycles, 3 weeks per cycle. However, the concentration of CEA (72.1 ng/ml) and CT images suggested that the curative effect was not that satisfactory, and it was found that the pulmonary lesions were partially absorbed, which was considered to be inflammatory or fibrotic lesions. So the adjuvant therapy was replaced by FOLFIRI regimen comprising irinotecan combined with calcium folinate and 5-Fluorouracil (5-FU), every 2 weeks as a cycle. Meanwhile, cetuximab was added to the regimen at a dose of 800 mg. A routine examination revealed that his CEA concentration decreased to 5.7 ng/ml without symptoms after using the FOLFIRI regimen and cetuximab for 24 cycles (Fig. 4). Nevertheless, because the patient suffered from myelosuppression, we discontinued this regimen. Four months later, chest and abdominal CT and MRI showed thickening of the distal intestinal wall of sigmoid colon, which was considered as tumor recurrence. Rechallenge of the cetuximab (800 mg) and FOLFIRI regimen (irinotecan 280 mg/dl, calcium folinate 1000 mg/dl, 5-FU 4.0 g CIV) was performed according to the suggestions of MDT discussion. The patient had severe myelosuppression after 2 cycles of chemotherapy, with leukocytes of 0.85 × 10^9^/L and platelets of 21 × 10^9^/L.

**Fig. 5:**
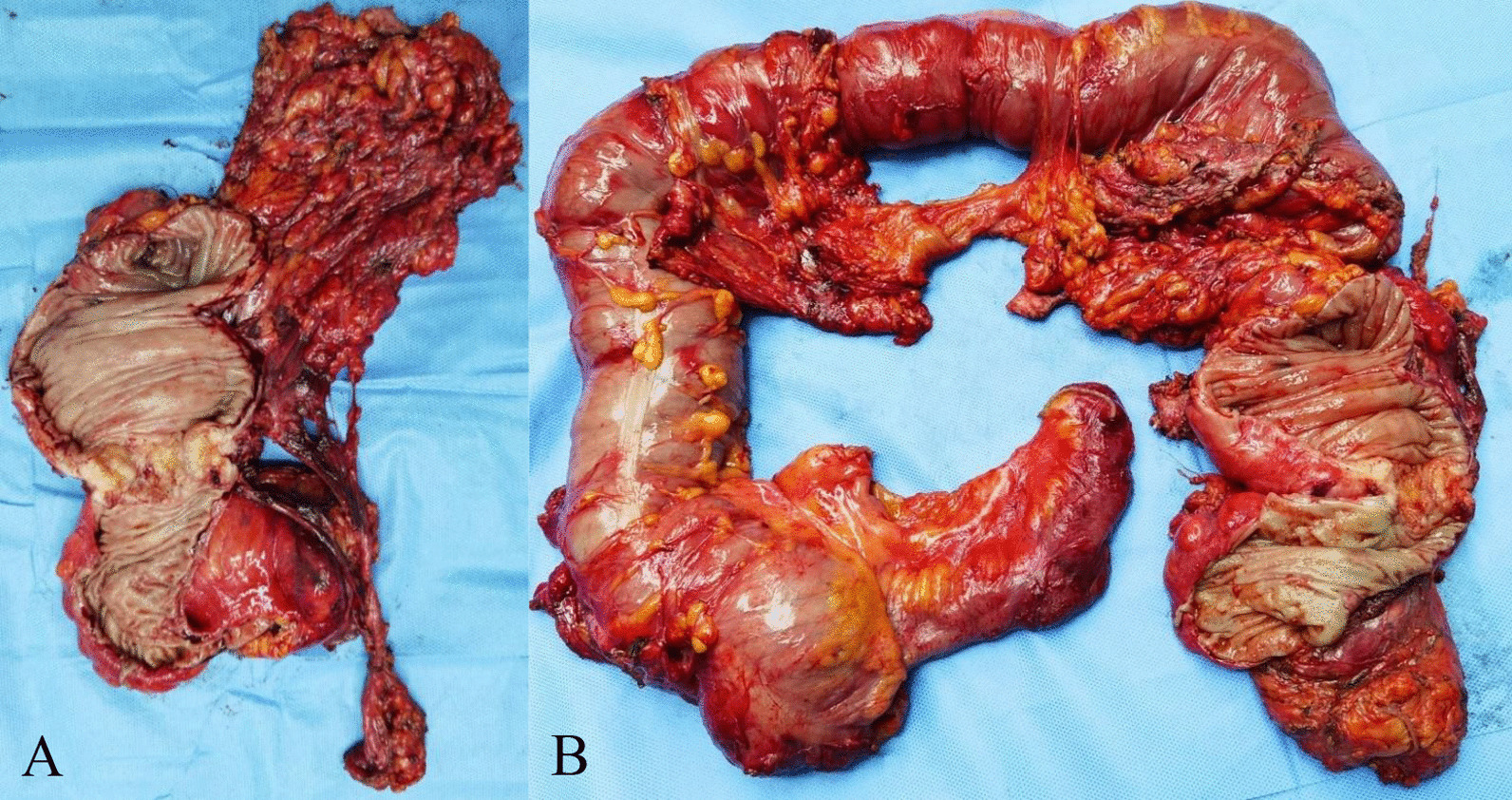
Postoperative gross specimen. The gross specimen clearly showed the primary focus of sigmoid colon

**Fig. 6:**
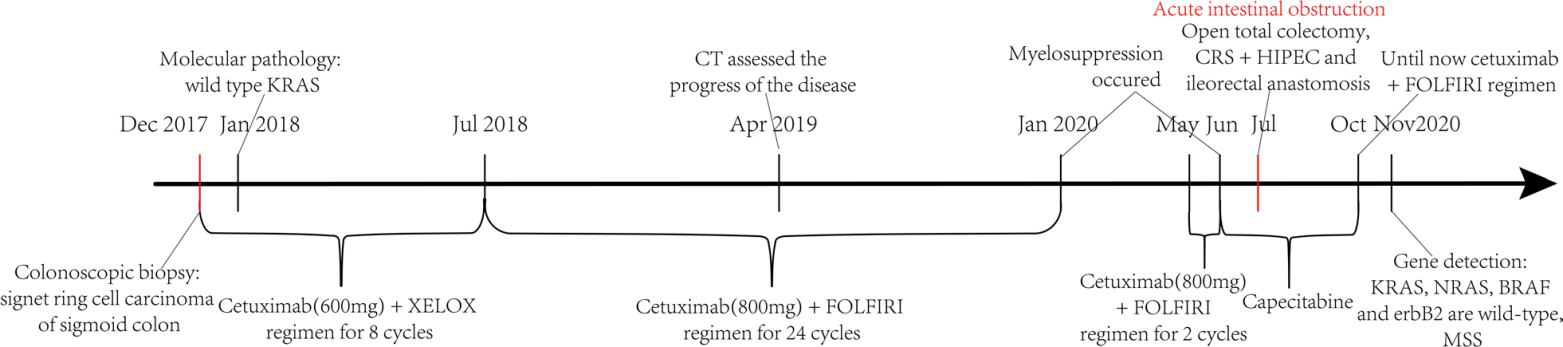
Clinical course over time. It includes the treatments, diagnostic procedures and timing of disease progression

In case of distant metastasis of tumor in the abdominal cavity, intraperitoneal thermal perfusion tube placement was performed, followed by intraperitoneal thermal perfusion chemotherapy combined with adjuvant chemotherapy. Following discussion at multidisciplinary team meeting, a recommendation for administration of FOLFIRI was made due to the patients metastatic status and good performance status. One month later, the patient developed acute intestinal obstruction, and was treated by intestinal obstruction catheter. After multidisciplinary discussion and the consent of the patient, he received HIPEC based on lobaplatin (60 mg, the rest remained unchanged), and the next day open total colectomy, CRS + HIPEC and ileorectal anastomosis were performed after intestinal adhesion was released. Figure 5 shows the primary focus of sigmoid colon (Fig. 5A) and the whole segment of colon removed during operation (Fig. 5B). According to sugarbaker CCR score [15], the CRS reached CCR0. After that, a large amount of warm normal saline containing lobaplatin was used to flush the abdominal cavity. After careful examination and hemostasis, the abdominal cavity was closed. The whole operation proceeded smoothly. After comprehensive evaluation of the overall situation of the patients, we implemented HIPEC. After 5 times of HIPEC (20 mg lobaplatin and 3 L normal saline at 43 °C for 60 min by specific dual circulation instruments), the tumor indexes showed a significant downward trend and the effect was good (Fig. 4). Molecular pathological examination was performed again, and no KRAS gene mutation was found. Gene detection reported that there was no mutation or amplification at KRAS, NRAS, BRAF and ERBB2, and it’s in microsatellite stable (MSS) status. The postoperative complications were slight leakage of anastomosis and chronic inflammatory polyps of digestive tract, and the conditions improved after symptomatic treatment. The treatment strategy was changed to capecitabine as a single drug and 3 months later, the cetuximab and FOLFIRI regimen was restored. After that, the patient has been in relatively stable condition, and no significant bone marrow suppression has occurred. By August 2021, the overall survival is more than 45 months, which displays significant curative effect (Fig. 6).

## Discussion and conclusion

The incidence and mortality rate of colorectal cancer rank first in digestive system malignant tumors worldwide. A clinical survey of 7 high-income countries shows that although the overall incidence rate of colorectal cancer in high-income countries tends to be stable or decreasing, the incidence rate in adults under 50 years is significantly increased [16–19]. Young patients with colorectal cancer are more likely to have tumor metastasis than elderly patients, and 22% of patients have distant metastasis colorectal cancer with the worst prognosis [20, 21]. The overall survival of patients with peritoneal metastasis of colorectal cancer is shorter, and the morphology of mucus cells or signet ring cells is associated with poor prognosis in patients with peritoneal metastasis. The presence of any signet ring cell differentiation is a recognized factor resulting in low survival rate [22–25]. For patients with left colon cancer, the overall survival of cetuximab + FOLFIRI group is 28.7 months and progression-free survival is 12.0 months. The overall survival of this case is 45 months, which is much longer than the median overall survival (6–25 months) of most clinical studies of peritoneal metastasis of colorectal cancer [5]. The additional survival time reflects the superiority of the treatment scheme in this case.

Previously, peritoneal metastasis of colorectal cancer was considered as advanced cancer and palliative treatment was the only alternative, but the proposal of HIPEC + CRS has brought about a change. Cytoreductive surgery can eliminate the visible cancer tissue in the peritoneum and abdominal pelvis. The thoroughness of surgical resection of CRS is an important factor affecting the prognosis of patients. Only by ensuring that the resection of CCR0 stage can achieve better clinical effect, which also limits the scope of application of the operation. Hopefully, preoperative neoadjuvant chemotherapy and transformation therapy including HIPEC can reduce the volume of the tumor, so as to meet the conditions required for CRS. And this case is a successful demonstration. In addition, although CRS + HIPEC is a common treatment for peritoneal cancer, its application is still controversial due to its high recurrence rate and toxic and side effects. Technical differences in different regions, selection of chemotherapeutic drugs, peritoneal perfusion time, whether HIPEC is combined with CRS and other factors may affect the final results [9]. Although a variety of clinical studies show that CRS + HIPEC benefits greatly, there is controversy in the academic community. But others show that HIPEC can not prolong the survival compared with CRS alone. They even demonstrated that CRS combined with oxaliplatin based HIPEC can not prolong the survival, but increase the adverse reactions [26, 27]. The risk of recurrence depends largely on the thoroughness of CRS. However, since lobaplatin has much less side effects than oxaliplatin, and from the clinical experience, the effect of CRS + HIPEC based on lobaplatin is indeed better than that of CRS alone, so we reasonably speculate that this may be due to the corresponding difference caused by the pharmacological effect of lobaplatin. And it is also reasonable to suspect that the difference in the reduction degree of CRS has a great impact on it, which obviously needs further research to confirm. Therefore, more factors should be considered in the formulation of treatment plan, including the location and genotype of primary tumor, which will help individualized treatment and enhance the curative effect.

At present, the most representative HIPEC drug is oxaliplatin, which is also commonly used in clinic. Previous review has shown that oxaliplatin based CRS + HIPEC has a high proportion of serious postoperative complications, so there is an urgent need for new generation of drugs to optimize the corresponding treatment [28]. Lobaplatin, the third generation platinum, has also been found to be able to be used in HIPEC. It is reported that the only side effect of lobaplatin is bone marrow suppression and it is equivalent to oxaliplatin but safer. However, if it is not administered intravenously but only used for intraperitoneal thermal perfusion, the side effect is mild. Therefore, in theory, intraperitoneal thermal perfusion with lobaplatin is safe and effective [29, 30]. In the majority of studies, lobaplatin was administered 50 mg/m2 or 40–60 mg at a time. But different from the conventional usage, we used 20 mg each time, once a day, continuous perfusion for 5 days. Many studies have shown that the efficacy of lobaplatin depends on its dose and its half-life is short. Although the single dose of lobaplatin is far lower than the recommended dose, multiple doses in a short time can achieve a certain degree of drug accumulation, so as to exert its efficacy in vivo [31]. Several studies have confirmed that lobaplatin is effective in the treatment of peritoneal cancer [32–34]. Although complications, such as anastomotic leakage and intestinal fistula, may occur after CRS with lobaplatin HIPEC, adverse events may be avoided as much as possible by studying the optimal dose and optimal perfusion time.

Another feature of this case is the realization of precise treatment with cetuximab at the genetic and molecular levels. EGFR signaling pathway plays an important role in the survival and proliferation of colorectal cancer cells. Cetuximab, a representative drug of EGFR monoclonal antibody, combined with chemotherapy is one of the first-line treatments for metastatic colorectal cancer. RAS and BRAF are classical molecular markers of mCRC, which are upstream and downstream in EGFR signaling pathway. Study has shown that Ras and RAF genotype play an important role in determining prognosis, and RAS wild-type gene has more survival advantage than mutant [35]. Therefore, whether their mutations occur is of great value for the efficacy evaluation. Moreover, the changes of HER2 amplification, PIK3CA and other driving genes also lead to the increase of EGFR monoclonal antibody resistance [36]. In our case, all of the molecular markers mentioned above are free of mutation, which support that the drug responsiveness can be good. Our case also confirms that the tumor is sensitive to targeted drugs, which is the major basis for the rechallenge of the previous regimen. As studies have confirmed that cetuximab rechallenge is effective in maintenance treatment [14], and considering that FOLFIRI is a first-line therapeutic drug, we made an audacious attempt and did not change the drug. Although drug side effects may occur, which might lead to drug withdrawal. These side effects can be related to the patient's physical condition and there is no evidence of tumor resistance. The early remission of the disease and the decline of tumor indexes indicate that the responsiveness of the medication is good. Even if there are adverse reactions such as bone marrow suppression in the later stage, the low-intensity transition scheme can be applied. After the patient's condition improves, the rechallenge of previous regimen still has the potential to treat tumors, and the original treatment scheme is discouraged to be abandoned prematurely.

In this case, not only HIPEC and CRS were performed, but also ileorectal end-to-end anastomosis was performed after total colectomy without fistula. According to the good condition of the broken end of the intestinal canal without obvious edema during the operation, we performed primary suture without fistula at the request of the patient. We believe that HIPEC without fistula is of positive significance to reduce the recurrence rate of tumor. Though slight anastomotic leakage occurred after operation, the situation improved through active treatment. We consider that the anastomotic leakage is mainly caused by HIPEC. How to reduce the occurrence of anastomotic leakage and other complications caused by HIPEC after CRS requires more clinical trials for in-depth research.

The disease progression is inevitable for patients with peritoneal metastasis of colon cancer, nonetheless, it might be successfully delayed through active and effective treatment. For peritoneal metastasis of colon cancer, preoperative neoadjuvant chemotherapy combined with CRS + HIPEC and targeted drugs can help the patients reach the state of No Evidence of Disease (NED), ameliorate the prognosis and prolong the survival time. In addition, the application of gene detection technology and the promotion of targeted drugs provide a valuable support for guiding clinical precision treatment [37, 38]. The positive attempt of lobaplatin and other new generation drugs may provide patients with better regimen options. Due to the high treatment cost, patient compliance and other practical resistance, it is difficult to obtain such long-term and regular follow-up and treatment data of CRS + HIPEC based on lobaplatin in the treatment of patients. Therefore, we only harvested this valuable case. Although the case may be useful for the selection of treatment for patients with peritoneal metastasis of colon cancer, the representativeness and universality of our treatment scheme need to be further confirmed by more clinical practice. This case suggests the potential clinical significance and bright prospect of lobaplatin in the treatment of gastrointestinal tumors by intraperitoneal thermal perfusion in the future. The above regimen is expected to become a new and effective treatment scheme, which could be widely used in patients with similar clinical conditions. However, the standardization of specific treatment dose and frequency also needs more detailed and in-depth research to determine the final clinical standard.

## Data Availability

The datasets used and analysed during the study are available from the corresponding author on reasonable request.

## Abbreviations

CA19-9: Carbohydrate antigen 19–9
CEA: Carcinoembryonic antigen
CRC: Colorectal cancer
CRS: Cytoreductive surgery
CT: Computed tomography
EGFR: Epidermal growth factor receptor
5-FU: 5-Fluorouracil
FOLFIRI: Folinic acid, fluorouracil and irinotecan
HIPEC: Hyperthermic intraperitoneal chemotherapy
mCRC: Metastatic colorectal cancer
MDT: Multidisciplinary team
MRI: Magnetic resonance imaging
MSS: Microsatellite stable
NED: No evidence of disease
PC: Peritoneal carcinoma
PM: Peritoneal metastasis
RAS-wt: RAS-wild type
XELOX: Xeloda and oxaliplatin
